# The Institutional Library

**Published:** 1918-11-02

**Authors:** 


### THE INSTITUTIONAL LIBRARY.

### THE FAIRY-TALES OF FIJI.

### Folklore from a Doctor's Note-Book.

TAKING for his subject the folklore and fairy-tales of the Lau Islands (Fiji), Dr. T. R. St.-Johnston has a delightful field, which he has explored lovingly.* Apart from his own personal interest in native legends and comparative mythology Dr. St.-Johnston had the privilege, for such it has proved to be, of an official appointment in the archipelago, and, as Commissioner of the Lau Islands and district medical officer there, the investigator's cards were all in his hands if he had the tact and the temper of culture necessary to play them wisely. This delightful book, the fragments spared by a tidal wave which was evidently jealous of the island secrets, is the proof that he was alive to his opportunity, and the interest which the author feels for the native legends communicates itself to the reader, an interest superadded to the charm of the tales themselves. Dr. St.-Johnston luckily takes more interest in the legends than in the interpretation which the anthropologist or comparative mythologist can place upon them, for he knows that these investigations too often have been disabled because, like Huxley, though with a difference, their interpretation, their theory, has been of more moment to such students than the mystic meaning of the legends, which, as a rule, is quite beyond their apprehension, though perhaps they can comprehend it in the rough. He is therefore wisely cautious in attempting to reconcile seeming contradictories (each of which, in the case of a myth, is usually merely an emphasis now on this and now on that "mystery," the final resolution of which will be achieved rather in eternity than in time). He is cautious, too, in offering explanations, partly because he respects the myth, because a myth is by definition but the outline of a truth "which it is not lawful to utter" to the general, and partly because he does not fall into the error of measuring its intelligibility by a European's power of comprehending it. These errors have been typical of much missionary effort, and the author naturally confesses himself fortunate to have found in the Lau Islands a place in which no resident missionary was stationed. The modern missionary's aim is usually to make his subject slough the old local beliefs, forget his traditions, and assume opinions—we cannot call them professions of faith—which, without the weight of tradition behind them, bemuse rather than enlighten the native, or any other, mind. The early missionaries were wiser, and attempted rather to graft Christianity on to the ancient tree of local tradition than to fell the tree in order to plant in its place a foreign acorn, the chances against the healthy survival of which were thereby scant indeed.

We dwell on the temper in which the material was collected and the mode in which the tales are told, because that temper and that mode alone can make the work of scientific, or indeed of literary, value. No man can understand that which he does not respect. The attitude of the true scientist is rather one of sustained receptivity than of impatient curiosity, for attention, that rarest of faculties in the investigator, is the reward of receptivity, just as what is pretentiously called active thought is the brink of the Gadarenean slope from which a herd of disordered notions rushes to the sea of its confusion. In fine, as a great man once remarked, "good thoughts are free children, and do not come by thinking." Myths are such good thoughts, in various degrees of apprehension, and he who would penetrate their mystery must become as they are if their secrets shall be revealed to him.

The Lau Islands lie about 200 miles east of Fiji, and their exposed position, which renders them a buffer to the Trade Winds, has made their shores the repository of drifting vessels, wrecks, and whatever wind and tide might hurry from the other Pacific islands nearer west. The islands themselves are rich in soil favoured by the coco-nut, and thus the prosperity of the copra industry in Fiji is in some sort dependent upon them. The islands are rich, too, in myth and *ethnos*, for the peoples of the Pacific have regularly migrated to them, and the student finds, as in the foundations of a buried city on which a modern town has been built, stratum under stratum of race, of customs, and of legend. To disengage the origin of either is a fascinating task, but the first step towards disentanglement must be a careful and unedited record of the fast-perishing traditions of the islanders. It is this which the author has attempted in part. It is the honesty of his attempt which gives to the book its value, and even the casual reader of fairy-tale and traditional story must be reminded that the degree of pleasure which he will experience in reading these pages is really due to that honesty alone. A myth cannot be retold. It is not susceptible of improvement. For the memory of a race is richer and profounder than the conscious art of any editor. An old tale is like the fruit of an ancient vine: touch but one grape and the bloom vanishes.

It would be a disservice to the author, therefore, to retell, even in outline, any of the stories which he has collected. The grave, the grim, the gay is in turn represented, and in each of the legends we feel rather that we are listening than that the focus of our thoughts is a page or two of print. The story of the rolling head, of the drum which would not sound to the smiter, of the six nights in which Tui Liku was tossed by demons and of his ingenious but unavailing efforts to escape—each of these, alike for its humours and its terrors, is

* *The Lau Islands (Fiji), their Fairy Tales and Folklore.* By T. R. St.-Johnston, M.R.C.S., L.R.C.P., Temp. Major, R.A.M.C. (Times Book Co., Ltd., 5s. net.)

### THE INSTITUTIONAL LIBRARY (*continued*).

**The Fairy Tales of Fiji** (*continued from p. 91*).
a good tale to be heard. The section devoted to the black magic of the islanders is also the setting for some dark stories, and for those in the two opposite camps there is enough to hold the ear of a child. Was it not Renan who defined the masses as those who seek to be amused? But in any case the daughters of memory, the Muses, are apt to respond, even to a casual invocation, so much more according to the measure of their own magnificence than to that of their votaries, that the tales which they offer can amuse those who seek for a-musement often almost as fully as they will haunt the ear of him or her who seeks the substantial felicity that abides in them. To either class of reader we commend "The Lau Islands," which the *Times* Book Company has been fortunate enough to publish.

**An Enquiry into the Analvtical Mechanism of the Internal Ear.** By SIR THOMAS WRIGHTSON, Memb. Inst.C.E. With an Appendix on the Anatomy of the Parts Concerned, by Arthur Keith, M.D., F.R.S. (London: Macmillan and Co. 1918. Pp. 254 + xi, and 9 plates.)

THE mechanism by which sound waves are conducted to the cochlea and there analysed is largely a problem in engineering to which Sir Thomas Wrightson has brought his expert knowledge, with the result that he has produced an entirely new theory of great importance, and which may be said to hold the field at the present time. Helmholtz's theory, that different fibres of the basilar membrane vibrate in resonance to different tones, has for some time been considered untenable, if only because the fibres are closely packed together and are not free to vibrate separately. The power of analysis possessed by the ear is very remarkable, for when different sets of periodic vibrations enter the ear the sensation of each tone is perfectly distinct. As the author says, when we consider the number of distinct pulsations which must vibrate in the ear in listening to the performance of a large orchestra, from which our ears receive numerous waves varying from twenty-five feet to three or four inches in length, and when we know that we can also at the same time hear other less musical sounds, such as the rustling of dresses, the whispers of the audience, and the distant sounds of the street, the mind is oppressed with the complication of the problem. The first part of the work is occupied by the graphic representation of compound sound-waves, and by a description of an instrument designed for their production, and is followed by a very careful description of the mechanism of the middle ear and cochlea. This leads up to the theory, which is briefly as follows. During the compression stage of the sound-wave the drum-membrane moves inwards and the pressure is passed through the ossicles to the much smaller area of the foot-plate of the stapes and thence to the labyrinthine fluid as in a small intensifying press. As the liquid is incompressible, the pressure passes instantaneously from the scala vestibuli to the scala tympani and fenestra rotunda, and the basilar membrane moves downwards. Corti's organ is thereby carried obliquely downward and the cilia of the hair-cells are bent by their movement relatively to the tectorial membrane in which they are imbedded. In the second phase of the wave this downward movement is reversed by a return to the position of rest; in the third phase, the negative or rarefaction stage, there is a movement of all the parts concerned in the opposite direction, and finally another return to the position of rest. There are thus four phases in the complete cycle, and the author postulates four impulses in every cycle, not only at each crest and hollow but also at each "crossing point" of the position of rest. In compound waves each change of angle produces a minute change of momentum and a smaller impulse, and the author shows that, if each of these impulses stimulates the auditory nerve-fibres, the most complicated of compound sound-waves can be analysed and distinctly perceived. The theory also explains the phenomena of differential and summational tones. The book has not been written all at one time and would have been made easier to read if the thesis had been clearly and concisely stated and the stages of the argument more orderly arranged; but the theory is a notable one and satisfies the requirements in a way that no previous theory has done. The appendix by Professor Arthur Keith is written from the point of view of comparative anatomy and physiology, and is most interesting. The work will repay careful study.

**How to Become a Woman Doctor.** By EMILY L. B. FORSTER, with a Foreword by W. J. FENTON, M.D., F.R.C.P. (London: Charles Griffin and Co., Ltd. Price 3s. net.)

THE author, or rather compiler, of this little booklet, sets forth in convenient compass all that need be known by the parent or prospective student of the various examinations to be passed, fees paid, etc., in order to qualify as a medical practitioner. The information is not in any respect novel, and could be obtained equally well from the Dean of any of the numerous medical schools now admitting women students. The question of the prospects of the woman doctor is touched on, but in so vague a manner as to leave the prospective student entirely in the dark as to what return she may expect from the very considerable capital outlay involved. Instead of a carefully considered analysis of the income to be expected from tuberculosis work, school inspection, or industrial appointments, which would be of real value, we are treated to generalities, such as: "Brains, pluck, and a love of hard work are the three most necessary assets of a doctor. . . . More and more every year do women make their own way —or stand still. . . With influence and money a girl may get a lift up, but nothing but 'herself' can keep her in the position she achieves." Whilst as a final *bonne-bouche* we are treated to the remarkable statement that "a woman holding the necessary qualifications to be registered as a General Medical Practitioner (*sic*) has every prospect of a successful professional career."

**Flourless Puddings and their Sauces.** By a COUNTRY RECTOR'S WIFE. (London: John Lane. Price 6d. net.)

The difficulty about flourless puddings is that they cannot also be sugarless. For establishments in which sugar is not all taken up with beverages these recipes, however, suggest plenty of variety, and, if not strikingly original, have the merit of requiring but little time and trouble to prepare.

